# Immunotoxicity of Carbon-Based Nanomaterials, Starring Phagocytes

**DOI:** 10.3390/ijms23168889

**Published:** 2022-08-10

**Authors:** Tereza Svadlakova, Drahomira Holmannova, Martina Kolackova, Andrea Malkova, Jan Krejsek, Zdenek Fiala

**Affiliations:** 1 Department of Clinical Immunology and Allergology, University Hospital Hradec Kralove and Faculty of Medicine in Hradec Kralove, Charles University, 50005 Hradec Kralove, Czech Republic; 2 Department of Preventive Medicine, Faculty of Medicine in Hradec Kralove, Charles University, 50003 Hradec Kralove, Czech Republic; 3 Department of Pathological Physiology, Faculty of Medicine in Hradec Kralove, Charles University, 50003 Hradec Kralove, Czech Republic

**Keywords:** carbon-based nanomaterials, graphene, carbon nanotubes, monocytes, macrophages, immunotoxicity, immunomodulation, inflammasome

## Abstract

In the field of science, technology and medicine, carbon-based nanomaterials and nanoparticles (CNMs) are becoming attractive nanomaterials that are increasingly used. However, it is important to acknowledge the risk of nanotoxicity that comes with the widespread use of CNMs. CNMs can enter the body via inhalation, ingestion, intravenously or by any other route, spread through the bloodstream and penetrate tissues where (in both compartments) they interact with components of the immune system. Like invading pathogens, CNMs can be recognized by large numbers of receptors that are present on the surface of innate immune cells, notably monocytes and macrophages. Depending on the physicochemical properties of CNMs, i.e., shape, size, or adsorbed contamination, phagocytes try to engulf and process CNMs, which might induce pro/anti-inflammatory response or lead to modulation and disruption of basic immune activity. This review focuses on existing data on the immunotoxic potential of CNMs, particularly in professional phagocytes, as they play a central role in processing and eliminating foreign particles. The results of immunotoxic studies are also described in the context of the entry routes, impacts of contamination and means of possible elimination. Mechanisms of proinflammatory effect depending on endocytosis and intracellular distribution of CNMs are highlighted as well.

## 1. Introduction

**Figure 1:**
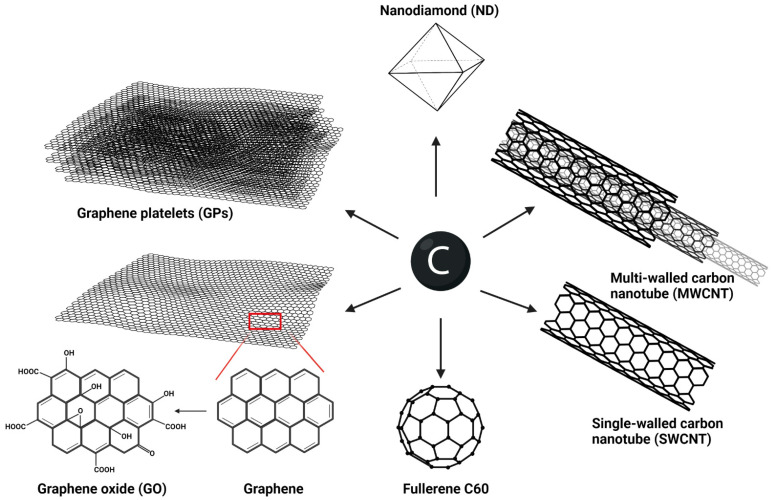
Overview of the most studied carbon allotropes that exhibit different structures and functions, created with BioRender.com.

Carbon is a fundamental element of all living matter on Earth. Carbon also represents the main building element of carbon-based nanomaterials and nanoparticles (CNMs; Figure 1). The first CNMs were discovered/prepared in 1980 and, since then, their number and potential use have constantly increased [1]. Currently, this heterogenous group of inorganic nanomaterials (NMs) includes amorphous particles, i.e., ultrafine carbon particles, carbon nanoparticles and carbon dots, as well as sp2 allotropes (nanotubes, graphene, fullerenes, carbon quantum dots) and sp3 allotropes, such as nanodiamonds and lonsdaleite [2]. The presence of pure carbon provides CNMs with high stability, exceptional mechanical properties, including strength, stiffness, and toughness, as well as thermal and electrical conductivity. For this reason, CNMs may find application in many branches of industry, e.g., in separation processes, water treatment, or electronics [3]. Among the most studied CNMs are graphene, carbon nanotubes (CNTs), nanodiamonds (NDs) and fullerenes.

Graphene, the main star of the CNMs family, was first isolated from graphite in 2004 [4]. It is a flat monolayer of sp2-hybridized carbon atoms mutually arranged in a two-dimensional (2D) matrix resembling a honeycomb. This unique arrangement, which includes delocalized π-bonds, gives graphene unusual electronic and conductive properties. Graphene is also one of the firmest materials ever, and may serve as a building material for other CNMs. By rolling and wrapping graphene, we obtain CNTs and fullerenes, respectively [5]. Pristine graphene is a hydrophobic material with tendencies to aggregate. However, its large surface area allows various functionalizations leading to the production of a great number of graphene derivatives which are applicable in many ways [6,7,8]. The most common derivatives include multilayered graphene platelets (GPs) and few-layer graphene (FLG), both in either pristine form or modified form with individual layers bound by van der Waals forces [9]. GPs are usually the main intermediate products of widely used methods of graphene preparation, i.e., Chemical Vapor Deposition (CVD) or mechanical or chemical exfoliation of graphite [4,10,11]. Fragments of GPs with lateral dimensions smaller than 100 nm form graphene quantum dots (GQDs) that possess extraordinary photochemical and photoluminescent properties that are potentially usable in bioimaging [12]. The most researched derivative is graphene oxide (GO), with better colloidal stability and increased reactivity due to the presence of oxygen groups (hydroxyl, carboxyl, epoxy groups) [13]. Heat, chemical, or electrochemical reduction of GO produces reduced GO (rGO) [14].

Carbon nanotubes are formed by the cylindrical arrangement of a single layer of graphene (single-walled; SWCNTs) or two and more layers of graphene (multi-walled; MWCNTs), with each type differing with respect to its physicochemical properties. According to the number of the layers, the diameter of CNTs is in the range between several nm and 100 nm, and the length can reach up to dozens of cm. The ends of individual tubes are usually capped by a half fullerene [15]. Similar to graphene, CNTs are firm and durable NMs with many options of functionalization, which makes them usable in industry, construction devices, and medicine [16].

Effective functionalization can be carried out in fullerenes, as well. These buckyballs consist of hexagonal, pentagonal, or heptagonal rings of carbon atoms. According to the number of these carbons, fullerenes are referred as C_70_, C_80_, etc., with the most common one being C_60_. Fullerenes are chemically and thermally stable molecules that can be dissolved in organic solvents [17].

Another intensely studied CNM is NDs, which represent a diverse family of nanoparticles highlighted for their inertness [18]. The size of each particle ranges from 3–5 nm to 100 nm and the diamond core (sp3 bonded carbons) is usually covered by a graphitic structure [19]. Due to its stable fluorescent and generally unique optical properties, it is another candidate for bioimaging application [20].

The last increasingly studied group of CNMs consists of carbon dots (CDs), whose common feature is a size below 20 nm. These quasi-0D CNMs can be distinguished as graphene quantum dots, carbon quantum dots, and carbonized polymer dots [21]. Generally, CDs are favored for their optical properties, including fluorescence, their stability in water, and low cytotoxicity [22,23,24]. CDs can also exist in many possible variations due to the abundant sources of carbon available for their preparation, which further expands the possibilities of their use [21].

Although CNMs share unique physicochemical properties and offer a wide range of potential uses, there are still concerns regarding their safety. Along with massive production, there is also an increasing risk of direct contact with living organisms and possible adverse effects on human health. This is especially true for their potential use in biomedical applications, cosmetics, and the food industry. Although no CNMs are currently authorized for use in medicine, studies show that their potential is enormous [25]. They could be an important aid in bioimaging, detection and treatment. They can serve as signal amplifiers in imaging, including EEG and ECG, biosensors for the detection of various compounds, toxins or substances of protein nature (antibodies, antigens), carriers for the transport of drugs and genes, treatment of antibiotic-resistant infections [26]. Very interesting is the possibility of creating biocompatible prostheses, stents or scaffolds from them, which would allow their use in regenerative medicine and tissue engineering [27]. In the cosmetics industry, fullerenes are frequently used, for example, in hair shampoos, moisturizers and anti-aging creams [28]. In the food industry, they could find applications as preservatives and antimicrobial compounds [29]. Therefore, it is likely that we will be exposed to CNMs in increasing manner. For this reason, CNMs rank among the most researched substances in toxicology, as well. A regular finding of both in vitro and in vivo toxicological studies is the increased production of inflammatory cytokines, pointing to changes in the activity of the immune system [30,31,32,33].

As a highly organized network of specialized cells and biomolecules, the immune system plays a leading role in the outcome of the interaction of nanomaterials (NMs) with a living organism. It represents a defensive barrier against invading pathogens and foreign particles. Immune surveillance, which includes patrolling, recognition, and fast elimination of infected, abnormal, or dead cells and tissue, is critical in maintaining homeostasis. Violation of homeostasis and interference with immune functions can lead to chronic abnormalities and subsequent disruption of the entire system. Since components of the immune system are present in the bloodstream, tissues and mucous membranes, mutual contact with CNMs is inevitable.

CNMs are mostly non-biodegradable NMs and can persist in the body for a long time [34,35]. On the other hand, surface functionalization, such as oxidation, provides possible sites for enzymatic degradation [36]. In both scenarios, immune components, notably phagocytes (monocytes, macrophages) and specific immune functions are always involved [37]. This can lead to immunotoxic effects, which include cytotoxic and undesired immunosuppressive or immunostimulatory reactions [38]. This review summarizes recent findings on these effects in the context of recognition, processing, and elimination of CNMs by components of the immune system.

## 2. Entering the Body

Unintentional exposure of humans to CNMs usually occurs during their manufacture and post-processing. There are various entry routes by which CNMs can get into the organism; the most common being inhalation and ingestion [39,40]. The subsequent distribution in the organism depends on several factors, which include physicochemical properties such as size, charge or functionalization, and properties acquired after contact with the biological environment [41]. An example is the formation of the protein corona, which consists of biological components (usually proteins) quickly bound on the surface of CNMs. The first layer, the so-called hard corona, includes high-affinity proteins that irreversibly bind to the surface of NMs. The second layer consists of low-affinity proteins that reversibly bind to proteins of the hard corona. The composition of this so-called soft corona is variable and depends on the actual local microenvironment [42]. The final composition of the whole protein corona is determined by the initial physicochemical properties of NMs, especially by their surface and charge. Binding of proteins can modulate these properties, which is reflected, for example, in a change in the hydrodynamic diameter and colloidal stability [43]. The interaction between CNMs and the organism is a dynamic process of continuous changes by which CNMs go through in various biological systems [44].

As for health risk assessment, inhalation of CNMs is considered the key route of unintentional exposure. It is also the reason that the proinflammatory potential of CNMs is often associated with pulmonary toxicity. Generally, larger particles (1–10 µm) mostly remain at the level of the bronchi and trachea, whereas smaller particles may reach deeper levels, like the alveoli and alveolo–capillary barrier. The components of lung surfactant (mixture of phospholipids, proteins, and ions) that are present bind to the surface of the CNMs, which may result in changes in the surfactant’s viscoelastic properties and contribute to the outcoming pulmonary toxicity [45,46]. Moreover, such modified CNMs come into contact with pneumocytes and alveolar macrophages, where they are either phagocytosed or remain trapped in the extracellular environment [34,35]. Findings of CNMs in the blood, liver, spleen, or kidneys during in vivo studies imply that CNMs eventually reach the bloodstream by penetrating either the alveolo–capillary barrier or the intestinal barrier after ingestion [47,48].

A significant risk is associated with CNTs and carbon nanofibers (CNFs), which have repeatedly been shown to cause pulmonary toxicity via induction of chronic inflammation and fibrotization of lung tissue, as recently reviewed by Bergamaschi et al. [49]. In 2014, the International Agency for Research on Cancer Working Group officially classified long rigid MWCNT-7 (manufactured by Mitsui, Japan) as a potential carcinogen (group 2B) [50]. In two occupational studies by Fatkhutdinova et al., the data confirmed that exposure to MWCNTs led to local inflammation. In the first study, workers exposed to MWCNTs had higher levels of tumor necrosis factor (TNF)-α, interleukin (IL)-1β, IL-6, IL-8, IL-4, IL-5, and interferon (IFN)-γ in the sputum. The authors did not confirm an increase in inflammatory cytokines in blood; thus indicating that the inflammation is limited to the lungs [51]. In the second study, exposure to MWCNTs increased the levels of IL-1β, IL-6, TNF-α, and KL-6 (Krebs von den Lungen-6; a marker of fibrogenesis in the lung) in the sputum. Furthermore, in the sputum of workers younger than 35 years, the level of transforming growth factor (TGF)-β1 was significantly elevated compared to unexposed controls and older workers [52]. Svehdova et al. analyzed blood samples of workers that were exposed to MWCNT aerosols for at least six months and non-exposed individuals. The results showed a higher expression of mRNA for proinflammatory cytokines and signaling pathways (IL-6, CXCL-2, MAPK) in persons exposed to MWCNTs [53]. The immunotoxic effect of MWCNTs was also studied by Vlaanderen et al. and Kuijpers et al. Vlaanderen et al. evaluated (in the first phase) serum immune markers and pneumo-proteins in 22 workers exposed to MWCNTs and 39 controls. In the second phase, they assessed protein levels in a subset of 10 exposed workers and 6 controls. In the first phase, the concentration of CXCL11, CCL20, IL-1 receptor II, and fibroblast growth factor increased while IL-16 and cutaneous T-cell-attracting chemokine (CTAC) decreased. In the second phase, the levels did not differ, except for an increase of CTAC. These results indicate that exposure to MWCNTs influenced the function of the immune system [54]. Kuijpers et al. additionally conducted a two-phase study and revealed that exposure to MWCNTs, especially at higher concentrations, led to elevated levels of CRP and adhesive molecule ICAM-1 in both phases [55]. As for the other CNMs, Tang et al. showed that occupational exposure to carbon black nanoparticles (CB) is associated with increased levels of serum TNF-α, IL-1β, IL-6, MIP-1β and CRP, and that these substances can induce the endothelial expression of adhesion molecules (VCAM, ICAM) and chemokines that act as chemotactic factors (CCL2, CCL5, and CXCL8), thus recruiting leukocytes into blood vessels and potentially increasing the risk of inflammatory vascular disease [31]. In the study by Berger et al., 26 healthy volunteers were exposed to carbon nanoparticles of 10 μg, 50 μg, or 100 μg. Nanoparticles in saline were instilled in the lungs (bronchial segments). Blood samples and bronchoalveolar lavage (BAL) were collected six hours after exposure. In the bronchoalveolar lavage fluid (BALF), there were no significant differences between the measured parameter levels among the different dosage groups. Conversely, in the blood samples, a dose-dependent increase in neutrophil count was detected. These results suggested that inhaled CNMs can trigger systemic inflammation [56].

In the case of biomedical application, CNMs are assumed to be delivered intravenously, which allows 100% biological availability. Individual particles might easily reach all components of both innate immunity, like circulating monocytes, granulocytes, components of the complement system, and adaptive immunity, like lymphocytes. Subsequent processing or translocation would depend again on the initial physicochemical properties and functionalization of used CNMs and formation of biocorona [57]. Several studies have pointed out that the majority of intravenously administered CNMs end up in the liver and spleen [58,59,60,61,62] followed by the lungs, kidneys, blood stream and heart [58,62,63,64]. Compared to occupational exposure, in most cases, there was a significantly smaller or no proinflammatory response logically due to designing or choosing CNMs to be more biocompatible and bioresistant, e.g., via modification of the surface by binding polymers such as polyethylene glycol (PEG). For example, Zhang et al. compared the in vivo toxicity of two oxidized SWCNTs dispersed in bovine serum albumin (oxSG-BSA) and PEG, respectively (oxSG-PEG). The authors found no difference between oxSG-BSA and oxSG-PEG in the levels of IL-6, IFN-γ, and TNF-α in plasma and tissue lysates of mice after NMs intravenous administration. Additionally, both results were comparable to that of the control mice [62]. In both cases, the presence of PEG and BSA helped “mask” CNMs from immune surveillance.

Considering the above-mentioned studies, it is evident that, as well as the shape, the surface composition of CNMs also plays a major role in recognition by the immune system, particularly by innate immunity. The specific contribution of a variety surface modifications or adsorbents together with physicochemical properties of CNMs in the context of innate immune response is discussed later.

## 3. Trojan Horse

Innate immunity is basically designed to sense any possible intrusion. Phagocytes possess a wide range of membrane and intracellular receptors called pattern recognition receptors (PRRs) that are designed to recognize molecular motifs of various pathogens, like microbe-associated molecular patterns (MAMPs) and pathogen-associated molecular patterns (PAMPs). Intracellular PRRs represent, e.g., NOD (Nucleotide-Binding Oligomerization Domain)-like receptors (NLRs), which, among other things, are part of the inflammasome. An example of membrane receptors is a group of glycoproteins called Toll-like receptors (TLRs), which include TLR3, TLR7, TLR8 and TLR9, which are anchored in the membrane of intracellular vesicles, and TLR1, TLR2, TLR4, TLR5, TLR6 and TLR10, which are anchored in the cytoplasmic membrane [65]. Depending on the type of agonist, activation of these receptors leads to an activation of relevant protein complexes and transcription factors (e.g., NF-κB) and consequently a respective immune response. A typical agonist is lipopolysaccharide (LPS), the part of the outer wall of Gram-negative bacteria, which is recognized via TLR4 and stimulates the secretion of proinflammatory cytokines such as IL-6 and TNF-α. The LPS represents the most common contaminant in various chemicals and on laboratory surfaces, and due to its thermostability, it cannot be easily removed [66]. Therefore, LPS easily comes into contact with NMs and has been found to bind on their surface through hydrophobic or electrostatic interactions [67,68]. Such a contamination could influence the results of immunotoxicity assays and, if unnoticed, it could lead to misleading conclusions concerning nanoparticle safety [69,70].

Several studies have proven that simultaneous exposure to the LPS and certain types of NMs may boost proinflammatory response [71,72]. Li et al. demonstrated that the LPS binding on nanoparticles may not only increase proinflammatory response but also alter the formation of biocorona by averting stable protein adsorption [73]. Not surprisingly, the presence of the LPS also changes the uptake of the NPs. In a recent study, J774.1 mouse macrophages engulfed MWCNTs and pristine graphene more effectively when incubated with the contaminated material than with the depyrogenated material. The data also showed upregulation of NLRC4 inflammasome, which is associated with the presence of bacterial flagellin. Moreover, the presence of contaminated CNMs resulted in increased cytotoxicity [70]. Any biological contaminant adsorbed on the surface of NPs can easily enter cells. The problem is that under normal circumstances, intracellular penetration of some biomolecules is limited. However, when adsorbed on the surface of NMs, they are phagocyted together with NMs, which in this way serve as “Trojan horses” [71]. The subsequent immune response may then differ from the classical scenario of “free” biomolecules. It has been confirmed that LPS is specifically recognized by cytosolic caspases 4 and 5, which are involved in non-canonical activation of inflammasomes in humans [74]. Non-classical inflammasomes are usually associated with intracellular presence of Gram-negative bacteria and endocytosis of contaminated NMs possibly mimic this event [75]. It also offers a plausible explanation of the observed increased cytotoxicity, since the non-canonical inflammasome pathway usually leads to cell death [70,75]. In a study focused on NLRP3 inflammasome activation, graphene platelets that alone caused no proinflammatory response in a THP-1 cell model increased NLRP3 activation when combined with muramyl dipeptide (MDP), another bacterial PAMP. The release of IL-1β, the main product of NLRP3 activation, was even significantly higher than after stimulation by the MDP alone [76]. Under normal circumstances, MDP activates NOD2 receptor and serves as either a first or second signal for NLRP3 assembly [77]. There is a question as to whether the stronger reactivity towards the combination of GPs with MDP was a consequence of increased internalization of MDP together with GPs or the cumulation of signals from NOD2 activation and undetected changes caused by GPs. Nevertheless, it leads to the conclusion that the formerly safe nanomaterial could cause significant harm in synergy with present PAMPs. For this reason, evaluation of biological contamination should precede any NPs health risk assessment.

To evaluate biological contamination of CNMs properly is not an easy task. Most methods focus on LPS as the most prominent contaminant. The commonly used limulus amoebocyte lysate (LAL) assay has been found to often interfere with various types of NMs [78]. The LAL assay is based on the LPS-induced coagulation cascade, resulting in the formation of a gel clot. However, oxidized MWCNTs were found to adsorb LAL zymogen, which resulted in the activation of coagulation in the absence of LPS [79]. False positive results were also found in the case of graphene oxide (GO), which interfered with the chromogenic variant of LAL assay [80]. The authors suggested using an indirect method based on the detection of TNF-α secreted by primary human monocyte-derived macrophages in the presence or absence of polymyxin B, LPS inhibitor. This test is, however, limited by the cytotoxic potential of NMs [80]. Using specific Toll-like receptor (TLR) reporter cell lines for non-cytotoxic CNMs could be a suitable alternative [81]. On the other hand, possible unspecific interaction between some CNMs and TLRs must be considered [82].

## 4. Recognition and Uptake

Phagocytosis represents an essential tool for both the elimination of foreign particles and reparation of damaged tissues. Professional phagocytes are also among the first cells to encounter NMs [83]. These cells, notably the monocyte–macrophage system, represent a highly plastic group of cells that can be found in the blood stream (circulating monocytes) and in various tissues (residential macrophages, dendritic cells). In addition to phagocytosis, their main functions are the presentation of antigen, production of cytokines, elimination of damaged cells and tissue, remodeling of tissue, etc.; thus, they play a central role in innate immune response [84].Therefore, studies on the immunotoxicity of CNMs usually focus on macrophages and macrophage-like cell lines, particularly human monocytic THP-1, notably THP-1-derived macrophages, murine RAW264.7 macrophages and j774a.1 macrophages. The uptake and cytotoxicity have also been studied on human pulmonary epithelial A549, as lungs are the most likely to be exposed to CNMs. The results confirm that CNMs are usually quickly engulfed via endocytosis, particularly phagocytosis and receptor-mediated endocytosis [30,85,86,87,88]. Additionally, graphene microsheets have been found to spontaneously penetrate through the cell membrane [89]. Penetration by mechanical cutting and macropinocytosis was observed for nanodiamonds (NDs) [90]. Less is known about CDs, which were found either free in cytoplasm or enclosed in lysosomes. In this regard, the decisive factors could be the size of CDs and aggregation state, as free CDs were found to enter the macrophages freely while aggregates were predominantly engulfed by clathrin-mediated endocytosis [91]. CNTs have been linked with the concept of incomplete or frustrated phagocytosis that occurs when cells attempt to internalize particles bigger than them, which usually ends with cell death [92,93]. The question remains how phagocytes recognize CNMs and whether the presence of these particles disrupts the function and viability of these cells.

As mentioned above, the recognition of potential danger is mediated via interaction of a variety of intracellular or surface receptors of phagocytes with molecular motifs typical for damaged cells (DAMPs), pathogens PAMPs or MAMPs. Contaminated CNMs might be recognized through these motifs, and thus be engulfed [70]. In the case of CDs, several studies have highlighted the importance of the carbon source and the synthesis method. For example, the CDs prepared using citric acid are usually internalized at higher levels by macrophages compared to CDs from different source [94,95]. Additional modifications influence uptake as well. Moreover, we must consider the specificity of a macrophage model that is used for experiments. Thoo et al. discussed in their study that observed preferential uptake of phenylboronic acid-modified CDs could result from binding to sialic acid, which is expressed on the surface of cancer cell line J774.1 [94].

Another important factor, which modulates the CNMs uptake is the presence of biocorona. An in vitro study by Duan et al. showed that BSA adsorbed on GO weakened the interaction between the phospholipid membrane of A549 cells and the surface of GO, which subsequently reduced its uptake [96]. These results are also in union with the findings of higher uptake of CDs by THP-1 macrophages in serum-free conditions [97]. In another study, the authors confirmed a significant decrease of cytotoxic effects of blood protein loaded GO [98]. A similar “stealth” effect was observed for bovine fibrinogen, gamma globulin and transferrin [98,99]. Moreover, blood protein coating reduced the cytotoxicity of SWCNTs on THP-1 and HUVEC cells [99].

On the other hand, in a recent study focused on MWCNTs, the presence of BSA increased uptake through the scavenger receptor SR-A1 [87]. Since native BSA does not interact with SR-A1, the authors suggested a significant conformational change that led to the uncovering of biding site. Nevertheless, the effect was semi-additive, as the key factor was shown to be functionalization of MWCNTs. In the preceding study, the authors compared pristine MWCNTs with two differently functionalized MWCNTs in the absence of sera and found that RAW macrophages and transfected CHO(mSR-A1) successfully engulfed carboxylated MWCNTs, but failed to uptake pristine and amino-functionalized MWCNTs. Moreover, the uptake of carboxylated MWCNTs impaired the distribution of SR-A1 receptors and decreased phagocytotic activity towards common SR-A1 agonists [86]. Upon addition of BSA, SC-A1-mediated uptake of pristine MWCNTs increased; however, it remained lower than the uptake of carboxylated ones [87]. Although adsorbed proteins modulate the binding, uptake, and cytotoxicity of CNMs, persistence of interactions between the cell surface and the nanomaterial itself plays a significant role. Pristine SWCNTs activated both TLR2 and TLR4 and induced the release of chemokines regardless of the presence of serum [82]. Based on molecular docking simulations, the binding appeared to be guided by nonspecific hydrophobic interactions [82]. These results are correlated with the results of another study showing that C60 fullerenes and SWCNTs might bind to some TLRs via internal hydrophobic pockets [100]. The study did not confirm similar results for GO [82]. In contrast to CNTs, graphene possesses a planar structure that provides significantly higher adsorption capacity [101]. High protein adsorption could simply increase the thickness of the GO sheets and therefore limits destructive membrane interactions and accessibility of GO surface area [99]. In addition, the presence of carboxyl and hydroxyl groups on the GO surface provides different options for bio-interactions.

An important concern relates to the interaction of nanoparticles with proteins of the complement system. Upon activation through one of the three pathways (classical, alternative, and lectin), cooperation of these plasma proteins results in opsonization of microbes or cells, recruitment of phagocytes at the site of intrusion and, in some cases, the formation of membrane attack complex (MAC), which causes lysis of the cell membrane. As a part of the biocorona, complement proteins could promote an uptake of CNMs as well. Their interaction with CNMs can also lead to inadequate activation of the proteolytic cascade, which results in disruption of homeostasis. One of the early studies focused on the interaction between CNTs and complement and confirmed the activation of the classical pathway via binding of C1q to the surface of SWCNTs and double(D)-WCNTs. The study also confirmed the activation of the alternative pathway but only in the case of DWCNTs [102]. Additionally, C1q has been shown to bind to NDs, causing their agglutination, without, however, activation of the classical pathway. Nevertheless, C1q attachment promoted phagocytosis by macrophages and subsequent cytokine release [103]. Wibroe et al. demonstrated that GO activated the whole complement cascade depending on oxygen content and form [104]. Another study evaluated GO-induced complement C3 cleavage (activation of the alternative pathway) depending on functionalization. Coating of GO with PEG significantly reduced both protein binding and C3-based activation [105]. Activation of the complement system by GO was also inhibited by the binding of complement factor H to its surface. Moreover, coating of graphene with factor H achieved better protection against complement activation than coating with serum albumins [106]. It follows that CNMs surface could be engineered to avoid unwanted immune response.

Polymer coating represents a common method to reduce protein adsorption and improve stability and biocompatibility. PEGylation was shown to be partially successful in reducing CNMs’ cytotoxicity, as previously reviewed [107,108]. In contrast to this, Luo et al. reported that small, PEGylated GO nanosheets caused activation of peritoneal macrophages and release of proinflammatory cytokines after internalization. The authors suggested that PEGylated GO sheets were preferentially adsorbed onto and partially inserted into the macrophage membrane, causing amplification of interactions with surface receptors [109]. Another study, which compared PEGylated GO, polyacrylamide-coated GO, polyacrylic acid-coated GO and aminated GO, reported that polyacrylic acid-coated GO induced the least cytotoxicity both in vitro and in vivo. The presumed reasons were differences in biocorona, as polyacrylic acid-coated GO contained a small amount of immunoglobin G, which is a well-known opsonin [110]. Khramtsov et al. compared monocyte response to GO sheets modified either with linear or branched PEG. They found that branched PEG provided better “stealth” properties for GO due to lesser protein adsorption and subsequent lower uptake by monocytes [111]. Given the above information, not only physicochemical properties of CNMs but also the type and modification of selected functionalization, including LPS content, must be considered.

## 5. Inflammation

**Figure 2:**
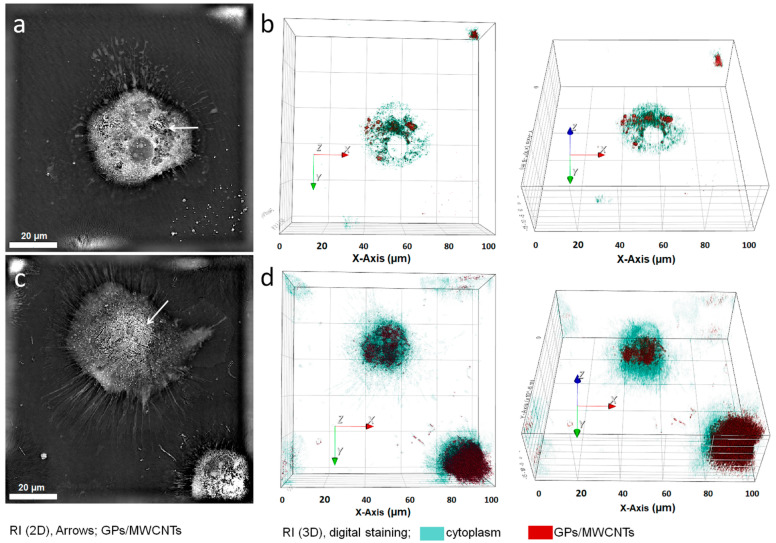
Macrophages are key cells in processing and elimination of CNMs. These representative live-cell images from a holotomographical microscope show human primary monocyte-derived macrophages cultured for 24 h with (**a**,**b**) 60 µg/mL GPs or with (**c**,**d**) 30 µg/mL MWCNTs. Primary monocytes were acquired according to protocol described in our previous work [81], and differentiated macrophages were maintained in RPMI 1640 wo phenol red, supplemented with 10% human autologous serum; RI–refractive index; Nanolive 3D Cell Explorer-fluo: Department of Clinical Immunology and Allergology, Faculty of Medicine in Hradec Kralove, Charles University.

The induction of inflammation is a key mechanism of NMs’ immunotoxic effect. Upon exposition to NMs, inflammation usually results from oxidative stress, the presence of contaminants and mechanical damage [112]. These effects also relate to CNMs’ interaction with an intracellular environment; thus, intracellular distribution of CNMs is a principal factor that alters the cell function. After internalization, CNMs are distributed depending on their properties as well as on their specific cell type. In the case of phagocytic cells, NDs and graphene flakes/platelets (GPs) are usually enclosed in endosomes/phagosomes (Figure 2a,b) [76,90]. On the other hand, internalization of CNMs by less efficient phagocytes or non-phagocytic cells might result in impairment of their intracellular homeostasis and thus cause cell death.

Carboxylated NDs were found to be more cytotoxic for B lymphocytes than for monocytes, despite significantly lower uptake by B lymphocytes. The authors of the study suggested better processing and clearance of NDs by phagocytic cells than B lymphocytes, which are known for their high sensitivity. Nevertheless, the uptake of NDs by monocytes resulted in the release of proinflammatory cytokines [113]. The THP-1 model showed that despite an accumulation of lysosomes, 100 nm NDs were able to cut through the lysosome membrane which was accompanied with the release of cathepsin B that activated the inflammasome [90]. Conversely, exposure to two types of pristine GPs initiated neither the inflammatory response nor a reduction in cell viability in human primary monocytes and THP-1 macrophages [76]. Similarly, there was no significant IL-6 IL-10 and TNF-α production after incubation of undifferentiated THP-1 monocytes with pristine GPs; however, there was a dose-dependent increase in the number of micronuclei, suggesting the genotoxic potential of those CNMs. The mechanism could be the possible interaction of GPs with naked DNA during THP-1 division [114]. Studies on 3D human lung models confirmed the absence of significant biological response under acute exposure scenarios [115]. Few-layered GPs did not cause any elevated release of proinflammatory cytokines from mouse bone marrow-derived macrophages and did not affect the viability and function of primary lymphocytes [116,117]. The pulmonary administration of GPs in rat model resulted in minimal inflammation, as well. The authors confirmed that inhaled graphene platelets were mostly ingested by macrophages without distinct lung pathology at the 1, 28 and 90 days post exposure [35]. These findings suggest that despite high accumulation in phagocytes, pristine GPs have an insignificant proinflammatory effect in general.

**Figure 3:**
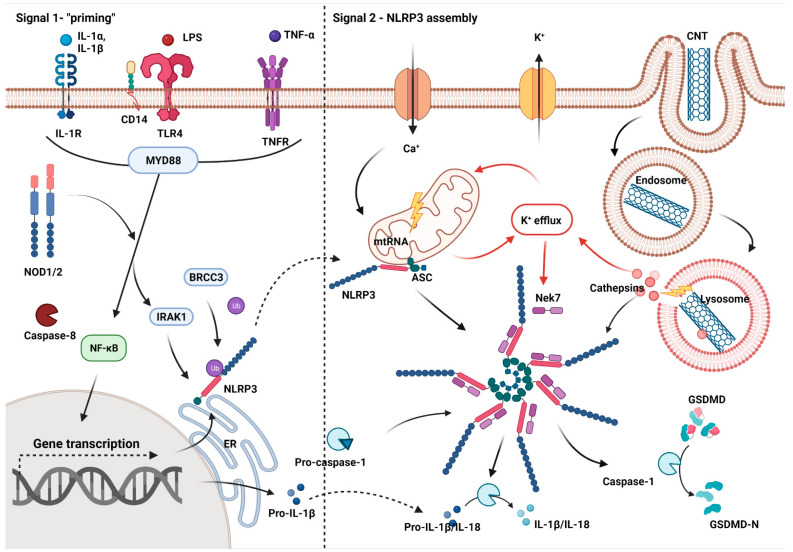
Mechanism of proinflammatory potential of CNTs: canonical activation of NLRP3 inflammasome in macrophages by disruption of lysosomes, created with BioRender.com.

Another situation occurs with CNTs, particularly long and rigid ones that are hardly expected to remain in closed vacuoles. Several studies have described the CNTs’ escape from endosomes, resulting in intracellular damage and acute inflammation associated with inflammasome assembly [118,119]. Inflammasomes are cytoplasmic macromolecular complexes that are evoked in response to infectious stimuli like whole pathogens or individual MAMPs as well as by cellular stress signals represented by sterile DAMPs. The result of activation is the cleavage of pro-caspase-1, pro- IL-1β, pro-IL-18, and pro-gasdermin D and, in some cases, proinflammatory cell death called pyroptosis [120]. NLRP3 (the nod-like receptor family pyrin domain containing 3) has the biggest role in association with NMs [121]. Activation of NLRP3 in macrophages by canonical pathway requires two signals. The first one induces the transcription of inflammasome components including pro-caspase-1 and pro-IL-1β. The second one includes a wide range of signals like DAMPs and activates NLRP3 assembly. In the non-canonical pathway, NLRP3 is activated through endogenous caspases 4 and 5 (caspase 11 in murine macrophages) which specifically bind LPS, triggering the release of ATP. This leads to massive formation of membrane pores and, due to the disruption of osmotic pressure, cell burst (pyroptosis) [122]. There is also an alternative pathway, which is typical for primary monocytes and can be activated by a single signal [123]. The typical ligand as well and the second signal for canonical NLRP3 is cathepsin B, leakage of which from damaged lysosomes was associated with MWCNTs (Figure 3) [119,124]. It seems that the proinflammatory potential of CNTs is predominantly related to high accumulation inside of phagocytes. Keshavan et al. compared the effect of three types of MWCNTs on macrophages and neutrophils. The uptake of long and rigid MWCNTs by THP-1 macrophages and human monocyte-derived macrophages resulted in inflammasome-dependent pyroptosis, while there was no uptake and cytotoxicity in the case of neutrophil-like HK-60 cells [125]. Similarly, there was no detectable uptake of MWCNTs by epithelial A549 cells compared to THP-1 and mouse NR8383 macrophages. Phagocytosis of MWCNTs by macrophages resulted in increased release of chemokines IL-8 and CXCL1, which was also confirmed in vivo [126]. Moreover, the results confirmed the predominant role of macrophages in processing of CNMs.

Despite activation of the inflammasome, damage of lysosomes by CNMs has also been associated with the disruption of autophagy [127]. Autophagy is a complex process that usually serves as a survival mechanism by removing misfolded or aggregated proteins, damaged organelles, and eliminates intracellular pathogens. It usually leads to modulation of inflammation and serves as an inhibitor of the inflammasome [128]. Fusion of lysosomes and autophagosomes is an important step in “healthy” maturation and degradation by autophagy flux but if dysregulated, e.g., via impairment of lysosomes, it might end in non-apoptotic cell death [129]. In the study by Wan et al., SWCNTs and GO induced formation of autophagosomes in murine peritoneal macrophages. Both CNMs, particularly GO, also accumulated in lysosomes which led to their destabilization and, consequently, to inhibition of autophagosome and lysosome fusion. The result was the blockade of autophagy and increased cell death [127]. Similarly, MWCNTs blocked autophagic flux in RAW264.7 model via lysosomal dysfunction associated with the downregulation of SNAPIN expression [130].

Induction of autophagy associated with increase of proinflammatory response was evaluated for GO in the study of Chang et al. They found that induction of autophagy was at least partially modulated by TLR4 and TLR9 activation. Increasing the concentration of GO led to high vacuoles accumulation and cell death [131]. Another study confirmed that GO caused TLR4-dependent necrotic death in mouse macrophages. High intracellular accumulation of GO caused cytoskeletal damage, oxidative stress and TNF-α release [132]. In contrast, endotoxin-free GO caused neither cytotoxicity nor proinflammatory cytokines release in human monocyte-derived macrophages. Moreover, GO suppressed the release of LPS-induced cytokines. In primed macrophages, the presence of GO caused inflammasome activation due to lysosomal damage which was probably caused by mechanical stress similar to that of CNTs [133]. Lipid extraction and/or oxidation by GO could also play an important role [134]. The direct effect of GO on lipid membrane was confirmed in isolated neutrophils. Interaction of GO with the cell membrane caused perturbations of plasma membrane lipids leading to the formation of neutrophil extracellular traps NETs. This study also confirmed that not all immune signaling is mediated via receptor; rather, the plasma membrane can behave as a sensor, particularly for solid structures [135]. Formation of NETs after exposition of mouse bone-derived neutrophils to micro-sized GO was also confirmed in another study. The authors also pointed out the importance of size, where nano-sized GO predominantly induced neutrophils degranulation [136]. So far, GO has been one of the most studied NMs due to its potential in nanomedicine. Despite the large quantity of available data, there are still contradictory results and information gaps, and it might be difficult to arrive at a uniform conclusion. Naturally, evaluation of contamination is as crucial as material characterization. Special attention should be paid to IL-1β production as inflammasomes appear to be a universal target of CNMs effect.

## 6. Modulation

**Figure 4:**
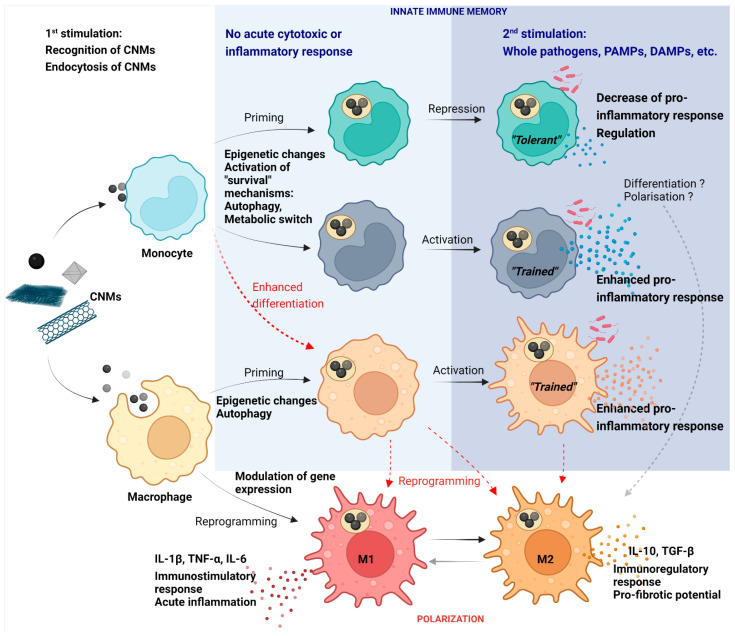
Schematic representation of immunomodulatory properties of CNMs in the monocyte–macrophage system. Without affecting cell viability, CNMs potentially induce innate immune memory via epigenetic reprogramming leading to metabolic switch and enhanced survival of cells. Along with the effect on the polarization state, CNMs modulate the resulting inflammatory response to various secondary stimuli. Created with BioRender.com.

The immune system works as a highly dynamic system, which in real life balances reactions to more than one stimulus by specific regulations. NMs that do not have acute cytotoxic or even pro/anti-inflammatory effects could still alter immune functions. This is especially true for CNMs which might persist in an organism for a long time. A good example is pristine GPs, which previously caused neither cytotoxicity nor acute inflammatory response in THP-1 and primary human monocyte model. However, their engulfment by monocytes under in vitro conditions not only led to better survival of cells, but also modulated their differentiation into macrophages and increased reactivity against bacterial stimuli [81]. Lebre et al. found that pristine graphene flakes similarly modulated bone-marrow-derived macrophages. They found that graphene promoted increased release of IL-6 and TNF-α against TLR agonists, presumably via a mechanism called innate immunity training [137]. This mechanism includes a non-specific, augmented immune response to a secondary stimulus and has previously been linked with metabolic and epigenetic changes in monocytes exposed to non-cytotoxic concentrations of particles or pathogens (Figure 4) [138].

A recent study on monocyte-derived macrophages and murine bone marrow-derived macrophages has observed that, without affecting viability, fragmented GPs modulated mitochondrial and respiratory capacity depending on macrophage polarization [139]. The ability of macrophages to polarize into proinflammatory M1 or anti-inflammatory M2 subpopulations is a highly regulated essential mechanism in the maintenance of physiological inflammation. Based on functional and electrophysiological measurements, authors suggested a preference for M2 polarization, and thus anti-inflammatory activity towards GPs [139]. Considering the potential of CNMs to activate the inflammasome via intracellular stress, preferential polarization towards M2 indicates an effort to balance emerging inflammation. Modulation of M1/M2 polarization has previously been studied for sublethal doses of several types of CNMs (Figure 4). While graphite nanofibers were found to cause M1 polarization in THP-1 macrophages, long, rigid CNTs triggered the expression of both M1 and M2 polarization markers and short MWCNTs triggered M2 polarization. All CNMs caused IL-1β secretion but without further polarization markers expression in the case of MWCNTs, which implicate the autoregulation mechanism [33]. Nevertheless, dysregulated, or prolonged polarization to M2 phenotype has a severe adverse effect. Zhang et al. evaluated the effect of SWCNTs and MWCNTs on mouse alveolar macrophages under M1 or M2 conditions. At the first stage, CNTs promoted the M1 phenotype, which was subsequently inhibited in favor of the M2 phenotype. In addition, the conditioned medium from exposed M2 macrophages boosted epithelial–mesenchymal transition and fibroblast-to-myofibroblast trans-differentiation via secreted TGF-β [140]. These results expand our understanding of the potential adverse effect of CNTs and their role in pulmonary fibrosis.

The exact mechanisms that precede the epigenetic changes leading to innate immunity training of monocytes/macrophages and reprogramming of macrophages caused by CNMs remain unclear. The modulation and enhanced differentiation of monocytes could also develop from autophagy, which is often associated with an effort to eliminate CNMs and has been found to be essential for monocytes survival and differentiation into macrophages [141]. Under physiological conditions, autophagy inhibits the inflammasome activation and release of proinflammatory cytokines, which, however, does not correspond with the often-observed augmented cytokine production [81,142]. Besides innate immune memory and autophagy, epigenetic changes are also linked with the reorganization of cytoskeleton which always occurs during endocytosis and subsequent processing of CNMs. It should not be excluded that endocytosis itself initiates signaling cascade leading to differentiation [143,144]. Moreover, the direct effect of CNMs on cytoskeleton has been confirmed several times already. For example, large GPs damaged cytoskeletal network in mouse macrophages and epithelial cells [89]. GO platelets disrupted migration of A549 and HeLa cells by reaction with actin [145,146]. The effect could be also indirect via mechanical oppression of intracellular components caused by high accumulation of CNMs.

Modulation of maturation and activity has been studied in dendritic cells (DC) as well. DC are indispensable antigen presenting cells that form a link between innate immunity and adaptive immunity. Their important function is enhancing specific T-cell responses, thus DC modulation could have several impacts on the whole immune system [147]. Yang et al. compared the effect of single layer GO and multi-layer GO on dendritic cell line DC2.4. They found that, whereas multi-layer GO was more cytotoxic and induced increased levels of ROS, single-layer GO caused cell aggregation but without significant cell death. Both GO stimulated production of TNF-α but no production of IL-6. On the other hand, single-layer GO elevated cytokine response to LPS by increasing release of both TNF-α and IL-6. On the contrary, IL-6 response by cells pre-treated by multi-layer GO was inhibited probably due to altered cell viability [148]. Effect of lateral size of GO was investigated by Zhou et al., who exposed mouse DC to micro-sized GO or nano-sized GO. According to their results, smaller GO was rather internalized, whereas larger GO adhered to cell membrane and induced cytoskeleton reorganization resulting in translocation of ICAM1, an adhesive molecule necessary for attachment of DC with T-cells. Subsequent cultivation with T-cells confirmed the formation of large clusters of DC-GO-T-cells, thus augmentation of T-cell activation [149]. Similarly, large GO induced maturation of human monocyte-derived DC via increased expression of costimulatory molecules CD80 and CD83 [150]. Compared to that, PEGylated GO with diameters under 200 nm inhibited expression of CD83 in human DC [151]. These results clearly indicate a significant role of size of GO and highlight interaction between GO surface and cytoplasmatic membrane.

The Immunomodulatory effect of CNMs was also evaluated in vivo using animal and disease models. Several studies have focused on allergies, especially hypersensitivity type I (IgE mediated). Park et al. exposed mice to different doses of MWCNTs. Analysis of blood and BALF revealed both an increased number of neutrophils and level of inflammatory cytokine such as IL-1β, TNF-α, IL-6, IL-4, IL-5, IL-10, IL-12, and INF-γ, respectively. The highest levels were those of IL-4, IL-5, and IL-10, which indicated an increased Th2 activity that activated B cell to produce IgE [152]. Comparable results were presented in the study by Inoue et al. Intratracheal instillation of both ovalbumin (OVA) and MWCNTs in mice led to the highest intensity of allergic inflammation and IgE production compared to other groups (placebo, OVA, MWCNTs) [153]. Nygaard et al. exposed mice to ovalbumin (OVA) and MWCNTs or SWCNTs. Both type of CNMs served as adjuvants increasing the intensity of the allergic response with elevation of OVA specific IgE [154]. Shurin et al. evaluated the effect of GO on the Th2-dependent immune response. In contrast to MWCNTs, GO attenuated Th2-type reactions (downregulated the production of IgE); however, it enhanced hyperreactivity and remodelation processes (goblet cell hyperplasia and smooth muscle hypertrophy) in airways in the murine model of asthma [155]. The results of these studies indicated that CNTs promote an allergic response in mice, while GO may attenuate these reactions but enhance the risk of irreversible remodelation of the airways. The MWCNTs also aggravated the chronic obstructive pulmonary disease (COPD) in the murine model. Beyeler et al. found out that the dose of 0.08 µg/cm^2^ of MWCNT administered by intratracheal instillation increased accumulation and activation of macrophages and dendritic cells in the lung parenchyma [156].

Soliman et al. showed that chronic exposure to MWCNTs is associated with an elevation in the number as well as an activation of alveolar macrophages, resulting in chronic pulmonary granulomatous inflammation and the formation of granulomas [157]. On the other hand, it should be mentioned that CNMs can also suppress inflammation. Dellinger et al. evaluated the effect of fullerene in a murine model of arthritis. The presence of fullerene in the affected joints after intraperitoneal administration led to attenuation of inflammation, reduced cartilage and bone erosion, and lowered the level of TNF-α [158]. Mai et al. used functionalized SWCNTs in murine models of acute and chronic graft-versus-host disease (GVHD). Acute and chronic GVHD increases the morbidity and mortality of patients after hematopoietic cell transplantation. It is vital to suppress the activity of immune cells. Intravenous administration of SWCNTs limited the proliferation of T and B cells and was associated with lower production of anti-host cytotoxic T cells and anti-host antibodies [159]. Tosic et al. proved that graphene quantum dots (GQD) inhibit neuroinflammation in rats with experimental autoimmune encephalomyelitis (EAE). Intraperitoneally administered GQD ameliorated the clinical symptoms of EAE, reduced central nervous system (CNS) infiltration with immune cells, attenuated the Th1 response, demyelination, axonal damage, and death of CNS cells [160]. CDs have been shown to have inhibitory effect in general. The leading mechanism is likely to be their antioxidant properties. CDs made of citric acid and glutathione successfully inhibited LPS-induced inflammatory response in J774A.1 macrophages by scavenging oxygen radicals and by downregulation of NF-κB and IL-12 production [161]. In another study, molasses-derived anionic CDs alleviated LPS-induced NO production in RAW 264.7 macrophages [162]. Ayaz et al. investigated CDs synthesized from carob and the influence of different surface passivation agents. They found that use of PEG or polyvinyl alcohol led to reduced proinflammatory response in RAW 264.7 model by inhibition of IL-6 and TNF-α production, while alginate increased the production of TNF-α, thus potentiated the proinflammatory response [23].

It is also necessary to consider capacity of CNMs to affect the composition and function of the microbiome (have an antimicrobial effect against certain types of bacteria and affect microbial diversity), which further influences the activity of the immune system and can be used, for example, in the treatment of inflammatory bowel diseases [163,164]. However, such modulation could also lead to disbalanced overpopulation of specific bacterial strain. The example is pristine graphene whose doses of 100 μg/mL increase the number of butyrate-producing bacteria (*Clostridium fimetarium*, *Clostridium hylemona* and *Sutterella wadsworthensis*) [165].

Taken together, the presented results suggest that CNMs have the capacity to modulate the activity of the immune system, both by increasing and decreasing inflammatory response. Special attention should be given another basic immune function like migration or phagocytosis. It is particularly essential for CNMs that are non-toxic and do not cause direct proinflammatory response.

## 7. Degradation

**Figure 5:**
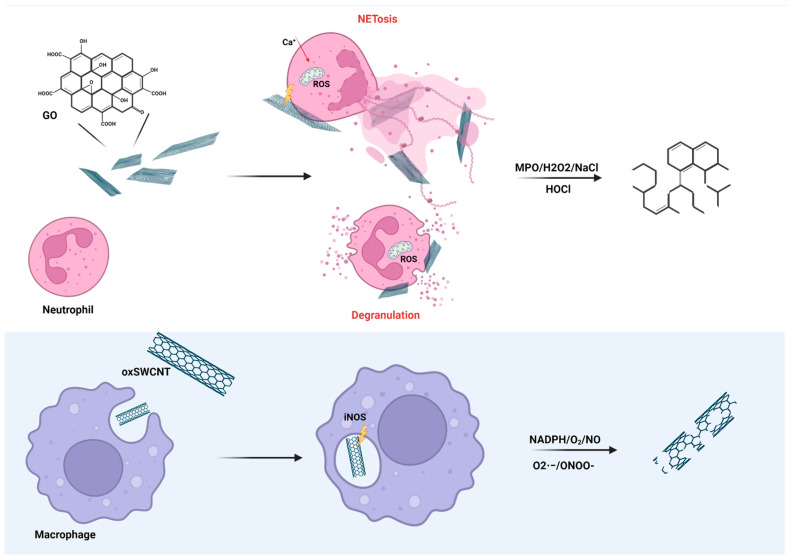
Schematic representation of potential enzymatic degradation of functionalized CNMs by neutrophils and macrophages. Neutrophils release NETs in response to GO with large lateral dimensions, while the contact with nanosized GO induces degranulation. Released myeloperoxidase (MPO) acts in sites of oxygen functional groups on the surface of GO. Similarly, enzymatic degradation of oxidized SWCNTs takes place in lysosomes by activity of NADPH, iNOs, and peroxynitrite (ONOO-), which are formed during the process. Created with BioRender.com.

Biodegradability is a crucial parameter for materials considered for nanomedicine. It determines the fate of particles in vivo. CNMs have always represented stable and persistent NMs; however, under specific conditions, potentially biodegradable with the assistance of immune cells. One of these conditions might be the atomic C/O ratio and hydrophilic nature. In a study by Kotchey et al., horseradish peroxidase was able to degrade GO but not reduce GO [166]. Similarly, myeloperoxidase (MPO), which is secreted by activated neutrophils (Figure 5), was able to degrade GO in the presence of a small amount of hydrogen peroxide. Biodegradation of GO was proportional to the percentage of carboxylic groups and aqueous colloidal stability [167]. Interestingly, in another study, production of MPO by human neutrophils incubated in whole blood occurred despite the presence of PEG on the surface of SWCNTs [168]. In an in vivo study, clearance of SWCNTs was significantly reduced in MPO knockout mice in contrast to wild-type animals suggesting effective degradation in living body and not only in vitro [169]. A recent study focused on the possible degradation of GO by eosinophil peroxidase EPO in the presence of hydrogen peroxide and NaBr. Despite being incomplete, degradation of GO samples occurred within 90 h of treatment [170]. This aligns with the study by Kagan et al., where human EPO and murine EPO from ex vivo activated eosinophils degraded oxidized SWCNTs [171].

The role of NADPH and oxidative burst in biodegradation of oxidized CNMs was confirmed in the case of macrophages (Figure 5). In another study by Kagan et al., oxidized SWCNTs were degraded by peroxynitrite from activated THP-1 macrophages. Moreover, the clearance of SWCNTs was significantly reduced in NADPH-oxidase-deficient mice [172]. Hou et al. compared biodegradation of pristine SWCNTs, oxidized SWCNT and OH-SWCNTs on RAW264.7 model. The respiratory burst in activated macrophages played an important role in the degradation of oxidized SWCNTs and OH-SWCNTs, whereas p-SWCNTs were resistant to biodegradation assumingly due to the lack of reactive sites for oxidative attack [173].

## 8. Conclusions

Like other NMs, CNMs represent a family of exogenous particles with a wide range of possible interactions with the immune system. Depending on their form, size, shape, functionalization, and purity, they can either directly trigger inflammation, which leads to disruption of homeostasis, or act indirectly by modulation of immune cell functions without altering cell viability.

As CNMs are mostly non-biodegradable and can persists in the organism for a long time, the possibility of potential immunomodulation is increasing. Special attention should be given to their effect on maturation and polarization of innate immune cells which are essential in initiation of the immune response. The presented results suggest that the shape and size are crucial properties influencing direct proinflammatory effects of CNMs as well as the subsequent behavior of immune cells. The immune system works as a highly organized network, and consequently disruption of one part may impact the whole system. We must consider that cumulation of CNMs in human body, especially in lungs, might have negative effect on the immune defense against common pathogens. On the other hand, with careful design and characterization of CNMs, their immunomodulatory properties are attractive for biomedical applications. These applications are based either on a direct effect of CNMs or the effect that CNMs have in conjunction with other materials or reagents when we use them as delivery platforms for drugs, biological imaging dyes, prostheses, etc. An example of the direct effect of CNMs is either reduction or amplification of inflammation in order to treat autoimmune disorders or cancer, respectively. There is also a non-negligible influence of CNMs on gut microbiota which modulates the immune system, as well. In the future, the knowledge of the immunotoxic and immunomodulatory effects of NMs could be of assistance in the development of tailored therapies, treatments of antibiotic-resistant infections, autoimmune diseases, or cancer. Unwanted toxicity might be reduced by the particular functionalization and well-targeted specific therapy.

Nevertheless, it is evident that there are still gaps in our knowledge. Furthermore, it is important to realize that different cell models vary in their sensitivity and ability to “sense” and engulf CNMs; the comparison between cancer cell models and primary cells may serve as an example. It is also important to distinguish whether these cells react on NMs alone or the molecules adsorbed on their surface. Taken together, the evaluation of immunotoxic potential of CNMs is an essential task, though not an easy one, and attention should be paid not only to proper characterization and contamination exclusion, but also medium composition. The addition of 3D models of human primary cells is also a necessary step, as we might easily miss the often overlooked but very important mutual influence in communication of cells.

## Data Availability

Not applicable.
